# Understanding Brassicaceae evolution through ancestral genome reconstruction

**DOI:** 10.1186/s13059-015-0814-y

**Published:** 2015-12-10

**Authors:** Florent Murat, Alexandra Louis, Florian Maumus, Alix Armero, Richard Cooke, Hadi Quesneville, Hugues Roest Crollius, Jerome Salse

**Affiliations:** INRA/UBP UMR 1095 GDEC ‘Génétique, Diversité et Ecophysiologie des Céréales’, 5 Chemin de Beaulieu, 63100 Clermont Ferrand, France; Ecole Normale Supérieure, Institut de Biologie de l’ENS, IBENS, Paris, F-75005 France; Inserm, U1024, Paris, F-75005 France; CNRS, UMR 8197, Paris, F-75005 France; INRA UMR 1164 URGI Route de Saint Cyr, Versailles, 78026 France; CNRS/UPVD UMR 5096 LGDP, 58 avenue P. Alduy, 66860 Perpignan, Cedex France

**Keywords:** Evolution, Genome, Ancestor, Synteny, Repeats, Polyploidization

## Abstract

**Background:**

Brassicaceae is a family of green plants of high scientific and economic interest, including thale cress (*Arabidopsis thaliana*), cruciferous vegetables (cabbages) and rapeseed.

**Results:**

We reconstruct an evolutionary framework of Brassicaceae composed of high-resolution ancestral karyotypes using the genomes of modern *A. thaliana*, *Arabidopsis lyrata*, *Capsella rubella*, *Brassica rapa* and *Thellungiella parvula*. The ancestral Brassicaceae karyotype (Brassicaceae lineages I and II) is composed of eight protochromosomes and 20,037 ordered and oriented protogenes. After speciation, it evolved into the ancestral Camelineae karyotype (eight protochromosomes and 22,085 ordered protogenes) and the proto-Calepineae karyotype (seven protochromosomes and 21,035 ordered protogenes) genomes.

**Conclusions:**

The three inferred ancestral karyotype genomes are shown here to be powerful tools to unravel the reticulated evolutionary history of extant Brassicaceae genomes regarding the fate of ancestral genes and genomic compartments, particularly centromeres and evolutionary breakpoints. This new resource should accelerate research in comparative genomics and translational research by facilitating the transfer of genomic information from model systems to species of agronomic interest.

**Electronic supplementary material:**

The online version of this article (doi:10.1186/s13059-015-0814-y) contains supplementary material, which is available to authorized users.

## Background

The Brassicaceae family is one of the major groups of the plant kingdom, being composed of more than 3600 species grouped in 321 genera assigned into 49 tribes [1–3]. Understanding the evolutionary history of extant genomes is the primary objective of comparative genomics research, fueled by the rapid increase in the availability of new genome assemblies. Ancestral genome reconstruction, the field of paleogenomics, delivers a common reference to study the evolution of descendants and is a natural and logical standpoint to obtain a chronological view of evolutionary processes. The Brassicaceae constitute a model clade for paleogenomics, given the availability of sequenced genomes such as *Arabidopsis thaliana*, *Arabidopsis lyrata*, *Capsella rubella*, *Brassica rapa* and *Thellungiella parvula.* These species represent the Brassicaceae lineages I and II, derived from a common ancestor ≈ 20 million years ago (hereafter mya), and include two closely related tribes: the Camelineae (*A. thaliana*, *A. lyrata*, *C. rubella*) and the Calepineae (*B. rapa*, representative of the Brassiceae, and *T. parvula*, representative of the Eutremae).

*A. thaliana* was the first plant genome to be sequenced [4], chosen partly for its phylogenetic relationship to agriculturally important *Brassica* species, thus greatly stimulating comparative genomics and evolutionary research in this family. Parkin et al. [5] first defined 21 genomic blocks (GBs) common to the *B. rapa* and *A. thaliana* genomes based on restriction fragment length polymorphism mapping data. Using genetic maps and comparative chromosomal painting (CCP) with pools of tens to hundreds of *Arabidopsis* bacterial artificial chromosome (BAC) clones arranged according to the genetic maps of *A. lyrata* and *C. rubella*, Schranz et al. [6] defined 24 ancestral GBs (A–X in Schranz et al. [6]; and re-evaluated in Mandáková and Lysak [7] and Cheng et al. [8]). They proposed that extant genomes evolved from an ancestral Brassicaceae karyotype (ABK) made of eight chromosomes. This scenario has since been refined, still using the 24 GBs but including the recently published *A. lyrata* [9], *C. rubella* [10], *T. parvula* [11], and *B. rapa* [12] draft genome sequences. The ABK (n = 8) was proposed to have evolved through pericentromeric inversions followed by reciprocal translocation mechanisms [13] into a proto-Calepineae karyotype (PCK) of seven chromosomes, which was proposed to represent the diploid ancestral genome of hexaploid *B. rapa* [8]. Schranz et al. [6] detected 61 of the predicted 72 (3 × 24) GBs in *B. rapa*, whereas Cheng et al. [8] more recently described 105 blocks or sub-blocks in this genome. Therefore, although ancestral Brassicaceae karyotypes (i.e., study of chromosome number variation) have been proposed, largely based on low resolution pairwise genome comparisons using the 24 GBs, a precise gene-based paleogenomic inference of ancestral genomes through robust protochromosome reconstruction and definition of ancestral gene order is still lacking. The inferred ancestral Brassicaceae genomes are necessary to unveil evolutionary changes (large-scale chromosome fusion, fission and translocation, as well as smaller-scale analysis of gains and losses of genes and functions, evolution of repeats, etc.) and assign them to specific tribes (Brassiceae, Camelineae, Eutremae) and species (*A. thaliana*, *C. rubella*, *A. lyrata*, *B. rapa* and *T. parvula*).

The impact of the lineage-specific paleohexaploidization event on the extant Brassiceae genome architecture has been the subject of intense debate recently [8, 14–16]. The triplicated Calepineae chromosomes reported in the extant *B. rapa* genome [17] experienced differential fractionation in gene content leading to the identification of three genomic compartments referred to as least fractionated (LF), medium fractionated (MF1) and most fractionated blocks (MF2), covering the ten modern *B. rapa* chromosomes [12, 14–16]. Such subgenome dominance (also referred to as partitioning) following whole genome duplication (WGD) has also been characterized in grasses leading to post-WGD dominant (D, reduced duplicated gene loss or LF) and sensitive (S, enhanced duplicated gene loss or more fractionated (MF)) subgenomes in paleo- or neopolyploids [18–21]. Genes retained in pairs or triplets after WGD in *B. rapa* tend to be enriched in functional categories such as transcriptional regulation, ribosomes, response to abiotic or biotic stimuli, response to hormonal stimuli, cell organization and transporter functions [22, 23]. Furthermore, genes located in the LF subgenome were proposed to be dominantly expressed over those located in the two proposed fractionated subgenomes (MF1 and MF2), suggesting that subgenome dominance in *B. rapa* acts at the level of both structures (retained ancestral genes in the D or LF compartment) and functions (dominant expression levels in this compartment [8]) of the considered genes. Single nucleotide polymorphism investigation at the population level between *B. rapa* accessions also tends to indicate a bias in mutation rates between D (or LF) and S (or MF) compartments, with genes located in LF compartments showing less nonsynonymous or frameshift mutations than genes in MF compartments [8]. Such a phenomenon following WGD complicates the identification of orthologous genes in *B. rapa* based on the *A. thaliana* genes. However, reconstruction of the Calepineae (or PCK) ancestral progenitors (i.e., with precise protochromosome structure and protogene content as well as orientation) would allow accurate elucidation of the fate of the paleohexaploidization event in Brassiceae in terms of extant genome architectures, largely unknown to date.

Here we report the gene-based reconstruction of a high resolution ABK and its two descendants, the Camelineae (ACK) and Calepineae (PCK) ancestral karyotypes. This is a prerequisite to investigating the evolutionary forces that have shaped extant Brassicaceae genomes at the chromosome (especially at synteny break points, paleo-centromeric regions, highly shuffled loci) and gene (especially between post-polyploidization LF and MF blocks) levels. Finally, the inferred ancestral Brassicaceae genomes allow accurate translational research of candidate genes for key applied agronomic traits from models to Brassicaceae crops based on a high-resolution evolutionary framework.

## Results

### Reconstruction and evolutionary fate of the Brassicaceae ancestral genomes

**Fig. 1:**
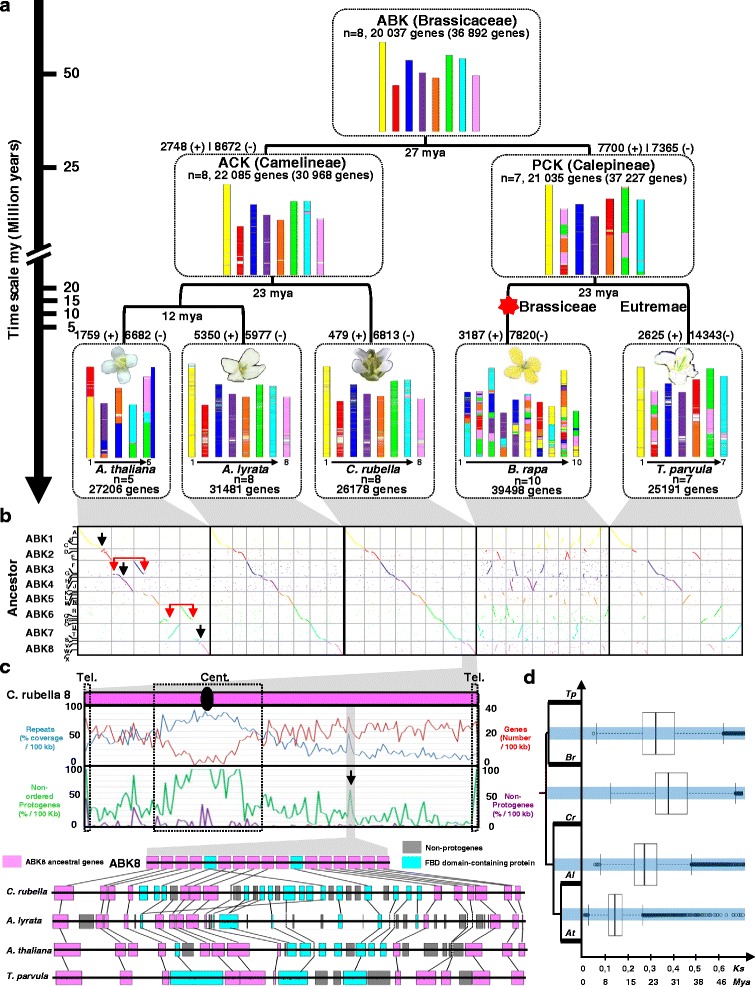
Brassicaceae ancestral genomes and evolutionary scenario. **a** The extant Brassicaceae genomes as a mosaic of colored blocks that illustrate the ABK1–8 protochromosomes. The number of chromosomes and genes in extant species and reconstructed ancestors (with the number of ordered protogenes followed by the total number of protogenes in parentheses) are given in the figure. The gene gain and loss characterized during Brassicaceae evolution are shown as “ *+* ” and “*-*”, respectively, on the tree branches associated with a time scale on the right expressed in millions of years (*mya*). The hexaploidization of *B. rapa* is illustrated with a *red star* **. b** Dot plot representation of the synteny between the ABK1–8 (color code and y axis) and the extant Brassicaceae (*A. thaliana*, *A. lyrata*, *C. rubella*, *B. rapa* and *T. parvula*; x axis). The 24 synteny blocks (A–X) according to the ABC system [6] are shown on the ABK1–8 (y axis) chromosomes. *Black arrows* and *red arrows* illustrate reciprocal translations leading to ancestral chromosome fusion with centromere loss (chromosome number reduction) or without centromere loss (no reduction in chromosome number), respectively. **c** *Top*: Densities (based on 100-kb physical windows) of annotated genes (*red curve*), repeats (*blue curve*), non-ordered protogenes (*green curve*) and non-protogenes (*purple curve*) for the extant *C. rubella* chromosome 8 (corresponding to ABK8, *pink*). *Bottom*: Microsynteny between *A. thaliana*, *A. lyrata*, *C. rubella* and *T. parvula* for a non-ordered protogene density peak highlighted with a vertical *black arrow* in (b). Conserved genes are linked with *black connecting lines*. Ancestral genes (on ABK protochromosome 8) retained in extant species are shown as *pink boxes* and non-ancestral genes as *gray boxes* and tandemly duplicated genes (i.e., FBD domain-containing protein) as *light blue boxes*. **d** Substitution rate (Ks) and dating in millions of years (*Mya*) (x-axis) of the speciation events (*blue horizontal lines*) between *A. thaliana* (*At*), *A. lyrata* (*Al*), *C. rubella* (*Cr*), *B. rapa* (*Br*) and *T. parvula* (*Tp*) (y axis) illustrated as dot boxes

Although an increasing number of genome sequences for Brassiceae species are available, the quality of assembly of only a few of them is sufficient to allow accurate evolutionary analyses at the whole genome level. For example, the availability of 59,102 non-assigned scaffolds of *Aethionema arabicum* was used in several gene-based evolutionary analyses [24], but is of limited use for large-scale synteny analyses aimed at reconstructing ancestral genome structures where scaffold assembly and anchoring onto chromosomes are necessary to avoid bias in ancestral genome reconstruction in terms of karyotype and gene content and order assessment. We thus selected the genomes of five sequenced extant Brassicaceae (*A. thaliana*, *A. lyrata*, *C. rubella*, *B. rapa* and *T. parvula*) and used phylogenetic relationships (including orthologs and paralogs, see “Materials and methods”) to reconstruct the ancestral genomes of the two major lineages (lineages I and II) of the family [25]. Previous analyses were based on 24 GBs, defined on the basis of *Arabidopsis* BAC contigs, and which therefore may at least partially reflect lineage-specific shuffling rearrangements that took place in *A. thaliana*. We used automatically reconstructed contiguous ancestral regions (CARs; also referred to as putative ancestral regions), which describe the order (gene-to-gene adjacencies) and orientation of ancestral genes, to reconstruct ancestral genomes. In a second step, manual CAR-scaffolding using an integrative approach based on the comparison of extant chromosome assignation led to the ABK and the PCK and ACK intermediates (Fig. 1a, see “Materials and methods”).

**Fig. 2:**
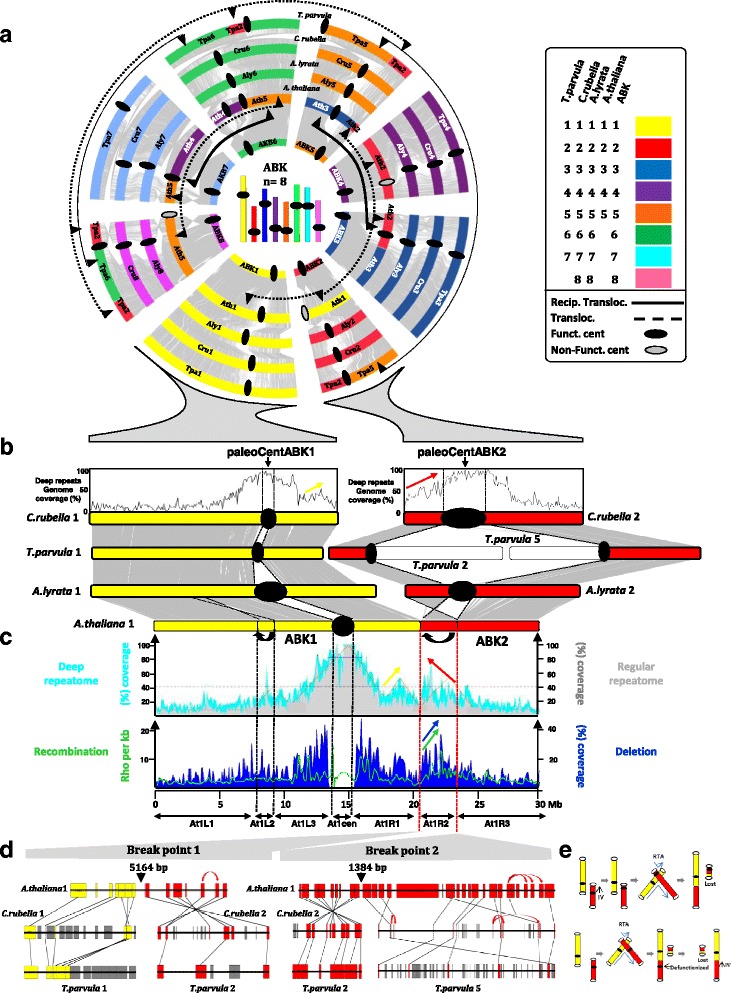
Distribution of repeats in the light of ancestral karyotype history. **a** Brassicaceae synteny. Schematic representation in circles of the *A. thaliana*, *A. lyrata*, *C. rubella* and *T. parvula* chromosomes according to a color code (illustrated on the *right*) highlighting the ancestral chromosome origins (ABK1–8, inner part of circle). Conserved genes between circles are linked with *gray connecting lines*. Functional and non-functional paleo-centromeres are illustrated as *black ellipses* and *gray ellipses*, respectively, on the chromosomes. Translocations and reciprocal translocations are illustrated as *plain* and *dashed black double arrows*, respectively. **b** Synteny between *A. thaliana*, *A. lyrata*, *C. rubella* and *T. parvula* regarding ABK1 (*yellow*) and ABK2 (*red*) protochromosomes where conserved genes are linked with *gray connecting lines* between extant chromosomes. The repeat density (deep/regular repeatome) is provided for *C. rubella* chromosomes 1 and 2 (*top*). **c** Deep repeatome (as percentage of TE coverage within 100-kb physical windows), regular repeatome (as percentage TE of coverage within 100-kb physical windows), recombination (Rho per kilobase), and deletion (as percentage of coverage within 100-kb physical windows) patterns on *A. thaliana* chromosome 1 (derived from the fusion of ABK1 and ABK 2, *top*) are shown as *light blue*, *gray*, *green* and *dark blue curves*, respectively. *Colored arrows* are as discussed in the text. **d** Microsynteny between *A. thaliana*, *C. rubella* and *T. parvula* at two inversion/translocation break points where conserved genes are linked with *black connecting lines*. Ancestral genes (from ABK1 and ABK2 protochromosomes) retained in extant species are shown as *yellow boxes* (deriving from ABK1) and *red boxes* (deriving from ABK2) and non-protogenes are shown as *gray boxes*. Local gene duplications are illustrated with *red arrows*. **e** Two scenarios of *A. thaliana* chromosome 1 evolution from ABK1 (*yellow bars*) and ABK2 (*red bar*s). *Empty circles* and *full circles* correspond to telomeres and centromeres respectively; *arrows* indicate inversions and *double arrows* translocations

The ACK, from which *A. thaliana*, *A. lyrata* and *C. rubella* are derived, is reconstructed here in eight protochromosomes (i.e., long CARs of conserved gene adjacencies) containing 22,085 ordered protogenes, with a separate set of 8883 non-ordered protogenes that are either singletons (no ancestral adjacencies could be inferred) or part of small CARs (less than 50 genes), defining a total set of 30,968 protogenes. *A. lyrata* and *C. rubella* have retained this eight-chromosome structure in their modern genomes, whereas the five *A. thaliana* chromosomes have evolved through three reciprocal translocations (driving the transition from eight ACK to five *A. thaliana* chromosomes with the loss of three mini-chromosomes containing three paleo-centromeres (Fig. 2e, top scenario) and two translocations between the eight Camelineae protochromosomes (black and red arrows in Fig. 1b, left dot plot). We reconstructed the *B. rapa* and *T. parvula* ancestral karyotype into seven protochromosomes containing 21,035 ordered protogenes (for a total of 37,227 protogenes, among which 16,192 are unordered or in small CARs). From this time point, *T. parvula* (Eutremae species) underwent no major shuffling events, retaining seven chromosomes, whereas *B. rapa* (Brassiceae species) experienced a paleohexaploidization and numerous structural rearrangements to give an extant genome structured in ten chromosomes (Fig. 1b; see details in the next section).

Finally, the most ancestral ABK is structured in eight protochromosomes containing 20,037 ordered protogenes (from a total of 36,892 protogenes, 16,855 of which are unordered). The parsimony of our method in reconstructing ancestral genomes is illustrated by the eight CARs from ABK containing 36,892 orthologous ancestral genes and covering 93 % (25,359 genes in ABK), 85 % (26,780 genes in ABK), 98 % (25,578 genes in ABK) and 92 % (23,257 genes in ABK), 92 % (36,415 genes in ABK) of the modern gene repertoire of, respectively, *A. thaliana* (27,206 genes), *A. lyrata* (31,481 genes), *C. rubella* (26,178 genes), *T. parvula*, (25,191 genes) and *B. rapa* (39,498 genes). The inferred ABK and associated robust orthologous relationships based on conserved gene adjacencies facilitate accurate transversal and non-biased calibration (Fig. 1d) for the timing of speciation events, with the ancestral Brassicaceae genome being older than 27 my (Fig. 1a). The reconstructed ancestral genomes are provided as Additional files 1, 2 and 3. Our data largely refine comparative genomics and genetics inferences of the Brassicaceae evolutionary history, previously based on 24 low resolution GBs that are now integrated into the reconstructed ABK1–8 chromosomes (Fig. 1b and Table 1).

**Table 1:** Comparison of the ABK and the extant Brassicaceae genomes of *A. thaliana*, *A. lyrata*, *C. rubella*, *T. parvula*, at the chromosome (1–8) and block (A–X) levels

ABK Chromosome	ABK Genes	AK System	ABC System	*A. thaliana* 5 chrs/27,206 genes	*C. rubella* 8 chrs/26,178 genes	*A. lyrata* 8 chrs/31,481 genes	*T. parvula* 7 chrs/25,191 genes
1	1661	AK2	A	AT1G01010/AT1G19835	Carubv10008547m.g/Carubv10008199m.g	918720/335233	Tp1g00060/Tp1g17630
1	1072		B	AT1G19840/AT1G36370	Carubv10010532m.g/Carubv10008652m.g	920937/922779	Tp1g17640/Tp1g30960
1	705	AK1	C	AT1G36380/AT1G56200	Carubv10010784m.g/Carubv10009198m.g	314057/924173	Tp1g31140/Tp1g41930
2	392	AK3	D	AT1G64690/AT1G57870	Carubv10022203m.g/Carubv10020358m.g	924446/475455	Tp2g00870/Tp2g05620
2	1339	AK4	E	AT1G65040/AT1G80950	Carubv10020264m.g/Carubv10022183m.g	315468/340237	Tp5g19570/Tp5g36040
3	2151	AK6	F	AT3G01010/AT3G25500	Carubv10016374m.g/Carubv10012854m.g	902874/905735	Tp3g00010/Tp3g23010
3	75		G	AT2G04039/AT2G07360	Carubv10014269m.g/Carubv10014847m.g	480058/480236	Tp3g23020/Tp3g24920
3	487	AK5	H	AT2G10950/AT2G20900	Carubv10013832m.g/Carubv10013473m.g	480278/480938	Tp3g26130/Tp3g34170
4	417	AK7	I	AT2G20920/AT2G26510	Carubv10023781m.g/Carubv10022938m.g	480941/481510	Tp4g00110/Tp4g07550
4	1825	AK8	J	AT2G26910/AT2G48160	Carubv10024573m.g/Carubv10024086m.g	481566/484008	Tp4g08450/Tp4g30080
5	211	AK9	K	AT2G01070/AT2G04038	Carubv10017082m.g/Carubv10018947m.g	484013/484308	Tp2g12540/Tp2g14790
5	310		L	AT3G25540/AT3G32940	Carubv10017681m.g/Carubv10018121m.g	484309/484755	Tp2g14800/Tp2g18310
5	353	AK10	M	AT3G44540/AT3G49890	Carubv10018745m.g/Carubv10018200m.g	897209/897870	Tp5g16660/Tp5g12220
5	1175		N	AT3G49900/AT3G63510	Carubv10017024m.g/Carubv10019526m.g	485350/486864	Tp5g12210/Tp5g00070
6	398	AK11	O	AT4G00026/AT4G05590	Carubv10003227m.g/Carubv10001670m.g	490066/943763	Tp6g04840/Tp6g00070
6	288		P	AT4G12620/AT4G06534	Carubv10001307m.g/Carubv10000223m.g	327036/490065	Tp6g10670/Tp6g04850
6	389	AK12	Q	AT5G30510/AT5G23030	Carubv10003425m.g/Carubv10001190m.g	489156/489679	Tp2g24110/Tp2g19950
6	1831		R	AT5G23000/AT5G01010	Carubv10000965m.g/Carubv10002949m.g	486867/894175	Tp6g41230/Tp6g22390
7	397	AK13	S	AT5G42110/AT5G32470	Carubv10004336m.g/Carubv10005331m.g	947127/494174	Tp7g07700/Tp7g05110
7	251	AK14	T	AT4G13480/AT4G16230	Carubv10006620m.g/Carubv10007255m.g	890633/493559	Tp7g14730/Tp7g11070
7	2161		U	AT4G16250/AT4G40100	Carubv10004014m.g/Carubv10004026m.g	490568/946646	Tp7g37630/Tp7g14760
8	437	AK15	V	AT5G47810/AT5G42670	Carubv10028554m.g/Carubv10026857m.g	494186/916060	Tp2g12470/Tp2g07130
8	1104	AK16	W	AT5G48340/AT5G60800	Carubv10026234m.g/Carubv10026078m.g	916323/496228	Tp4g08450/Tp6g22370
8	608		X	AT5G60830/AT5G67640	Carubv10028041m.g/Carubv10026089m.g	950837/496963	Tp2g24160/Tp2g30300
TOTAL	20037						

The inferred ancestral gene complements provide an immediate reference point to decipher gene gains and losses along the branches to the descendant genomes. On average, genes have been gained at a rate of 219 genes per million years, but lost at more than twice the rate, 541 genes per million years, with extreme variations between lineages (Fig. 1a). We examined how the reconstructed gene adjacencies in the ancestors may inform estimation of rates of local rearrangements that took place in the course of evolution in shaping the modern Brassicaceae species. A high rate of rearrangements during evolution will correlate with a loss of conserved gene adjacencies between the modern species, and thus with a higher proportion of singletons and small CARs (i.e., referenced here as non-ordered protogenes) in the reconstructed ancestors. Following this methodology and by comparing the extant *C. rubella* genome with its reconstructed ancestor, it clearly appeared that telomeric and centromeric regions of extant chromosomes coincide with higher numbers of *C. rubella* genes not included in the ordered protogene set (referred to as non-ordered protogenes in Fig. 1c, top) and which are, therefore, subject to considerable shuffling during evolution. Extensive rearrangements and local duplications have thus disrupted local gene adjacencies along *C. rubella* chromosome arms, resulting in local clusters where many ancestral genes could not be inserted into long CARs of the ancestral karyotypes (Fig. 1c, bottom). Interestingly, such local shuffling of loci also coincides with low gene densities and high transposable element densities in the extant *C. rubella* genome (r^2^ = 0.785 for transposable elements versus singletons in 100-kb windows). These results suggest that gene rearrangements in Brassicaceae have occurred in a non-random fashion, being concentrated at the extremities of chromosome arms (adjacent to telomeres and centromeres) and in specific loci where they are sources of synteny decay, making it more difficult to reconstruct ancestral gene content.

In conclusion, the Brassicaceae are derived from a diploid ancestor structured in eight protochromosomes containing 20,037 ordered protogenes that evolved over the last 27 my into extant species through enhanced structural plasticity at centromeres and telomeres as dynamic (duplication/inversion) loci.

### Evolutionary fate of the Brassicaceae paleo-centromeres

The mechanisms driving paleo-centromere loss during chromosome number reduction from ancestral to modern karyotypes and responsible for maintaining functional monocentric neo-chromosomes remain largely unknown. The reconstruction of the three ancestral genomes presented here greatly facilitates the identification of syntenic relationships between extant genomes, which are signatures of shared ancestry that can be illustrated as concentric circles (Fig. 2a, gray connecting lines and color code; Table 1). This representation immediately reveals two important features. Firstly, paleo-centromeres (highlighted by the synteny erosion on the circles) are retained at orthologous positions in extant Brassicaceae species, corresponding to the functional centromeres in the eight chromosomes of *A. lyrata* and *C. rubella.* Secondly, ancestral centromeres are lost in the remaining extant Brassicaceae after ancestral chromosome fusions/translocations (plain and dashed arrows in Fig. 2a).

We investigated the fate of the eight ancestral Brassicaceae paleo-centromeres through chromosome fusions and rearrangements leading to the five extant monocentric chromosomes in *A. thaliana.* Fig. 1b illustrates *A. thaliana* chromosome 1 that results from the fusion of ABK1 (yellow protochromosome in Fig. 2b) and ABK2 (red protochromosome in Fig. 2b) and two inversions (indicated with black arrows). Overall, *A. thaliana* chromosome 1 can be simplistically modeled into seven different evolutionary compartments (deriving from the fusion and the two inversions), At1L1, At1L2, At1L3, At1cen, At1R1, At1R2 and At1R3 (Fig. 2c, bottom).

The synteny observed between *A. lyrata*, *C. rubella* and *T. parvula* for ABK1 and ABK2 allows precise identification of orthologous paleo-centromeres, illustrated as black ellipses and called paleoCentABK1 and paleoCentABK2 in Fig. 2b. Our enhanced detection of potentially ancestral repeats, the “deep” repeatome (see “Materials and methods”), reveals regions of significant repeat density in addition to those defined by the centromere–pericentromere spaces (At1cen in Fig. 2c). The locations of paleo-centromeres on *C. rubella* chromosome 1 correspond to the highest transposable element densities. Examination of the regular and deep repeatome landscape of *A. thaliana* chromosome 1 (Fig. 2c, gray and blue curves) shows that the extant centromere (At1cen) corresponds to paleoCentABK1. Following the ABK1–ABK2 fusion leading to *A. thaliana* chromosome 1, monocentry was re-established by the retention of the (peri)centromeric structure and function from paleoCentABK1 and the loss of paleoCentABK2 (Fig. 2b, c).

The At1R2 compartment, taking into account the fact that it is a pericentric inversion, shows a similar increasing density of repeats, which may represent remnants of paleoCentABK2, when compared with the orthologous pericentromeric locus from *C. rubella* 2 (Fig. 2c, red arrow). The At1R1 segment also projects onto an orthologous repeat-dense region in *C. rubella* (Fig. 2c, yellow arrow), suggesting that significant repeat density in this region may be an ancestral characteristic of the Brassicaceae karyotype. Detailed analysis of the microsynteny between *A. lyrata*, *C. rubella* and *T. parvula* at the two breakpoints flanking the inverted/translocated region At1R2 led to the identification of 5164-bp and 1384-bp intervals in which no remnants of telomeric or centromeric repeats were detected, suggesting that paleoCentABK2 was integrally deleted during ABK1–ABK2 fusion (Fig. 2d).

In order to investigate the consequence of the fusion (also referred to as pericentric inversion followed by reciprocal translocation; see the next section) of two homocentric ancestral chromosomes, ABK1–ABK2, leading to the extant *A. thaliana* chromosome 1, we investigated patterns of structural variations (i.e., deletions found across 80 Eurasian *A. thaliana* accessions [26]) and recombination (i.e., map of crossover frequency from the same study) at the population genetics level. When comparing the deletion profile observed for subtelomeric regions of the *A. thaliana* chromosome 1 short arm (At1L1 and At1L2) and long arm (At1R2 and At1R3), we observed that deletions are more frequent in the At1R2 segment (Fig. 2c, blue arrow) compared to other regions (Fig. 2c), suggesting that this region, corresponding to the inverted ABK2 segment, is prone to deletions. The recombination profile shows an overall increasing crossover frequency from telomeres to pericentromeres, suggesting a correlation between repeat density and crossover frequencies, although the centromere itself is crossover suppressed. However, some megabase-scale peaks in crossover frequencies that apparently do not correlate with repeat density were also reported along *A. thaliana* chromosome 1 [27]. Interestingly, when comparing the different *A. thaliana* chromosome 1 compartments defined above, we observed that At1R2 presents the highest recombination rates (Fig. 2c, green arrow).

Two distinct molecular mechanisms (Fig. 2e) have been reported to explain centromere loss after ancestral chromosome fusions [13, 28]. In the first scenario (Fig. 2e, top; adapted from Lysak et al. [13] and Wang et al. [12]) a pericentric inversion, involving the entire short arm of ABK2 and a reciprocal translocation (RTA) between the short arm end of ABK1 and the pericentric end of the inverted ABK2, resulted in the fused *A. thaliana* chromosome 1 (without remnants of ABK2 centromeric sequences) and the loss of the small translocation product that contains the entire centromere of ABK2 (plus two chromosome ends). In a second scenario (Fig. 2e, bottom; adapted from Wang et al. [28]), *A. thaliana* chromosome 1 was alternatively produced by end-to-end joining through reciprocal translocation between ABK1 and ABK2, followed by ABK2 centromere defunctionalization, a largely speculative or at least unknown process, and finally ABK2 pericentric inversion.

Overall, chromosome number reduction from ABK to extant Brassicaceae species occurred through ancestral chromosome fusions (more precisely reciprocal translocations) where monocentry of the fused chromosomes is recovered through pericentric inversions that may promote higher local recombination and deletion rates.

### Fate of the ancestral karyotypes following polyploidization

**Fig. 3:**
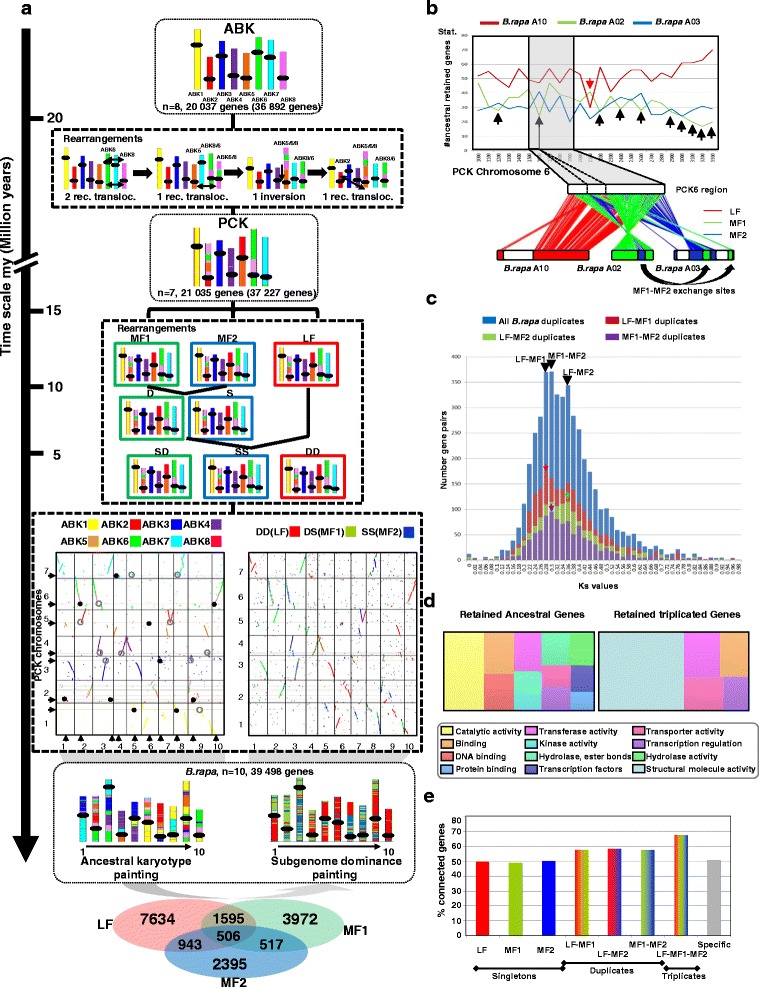
Impact of hexaploidization on the extant *B. rapa* genome architecture. **a** Evolution of the extant *B. rapa* chromosomes from the ABK1–8 (*top*) and PCK1–7 (*center*) protochromosomes. Evolutionary rearrangements in the course of the *B. rapa* paleohistory are detailed in *dashed boxes* according to a time scale on the right expressed in millions of years. Dot plots of the gene conservation between PCK (y-axis) and *B. rapa* (x-axis) are provided with a color code reflecting the ABK1–8 protochromosomes (*left*) and the LF–MF1–MF2 subgenomes (*right*), with *black arrows* indicating the modern centromere positions. Retained ancestral centromeres are illustrated as *plain black dots* whereas lost ancestral centromeres are shown as *gray open circles*. The extant *B. rapa* chromosomes (*bottom*) are illustrated as a mosaic of colored blocks reflecting the ancestral chromosomes (*left*) and the subgenomes (*right*) based on the number of ancestral genes retained as singletons, doublets or triplets (Venn diagram), thus defining the LF, MF1 and MF2 compartments. **b** The number of ancestral genes (from a PCK chromosome 6 region) within 100-gene windows on the post-hexaploidization extant chromosomes 10, 2, and 3 from *B. rapa* (*top*). The microsynteny locus between PCK6 and the extant post-hexaploidization relatives is highlighted in *gray*. The *B. rapa* chromosome 10 fragment appears entirely dominant (*red*) whereas *B. rapa* chromosomes 2 and 3 show exchanges of dominance/sensitivity (*green* and *blue* for MF1 and MF2, respectively). **c** Substitution rate (Ks, x- axis) distribution curves of gene pairs (number on the y-axis) for all detected pairs (*blue*), pairs located on LF–MF1 (*red*), LF–MF2 (*green*) and MF1–MF2 (*purple*) compartments. Ks distribution peaks are shown with colored arrows as described in the main text. **d** Gene ontology (GO) analysis of retained ancestral genes (*left*) and retained triplicated/duplicated genes (*right*). GO categories significantly enriched compared with the annotated *B. rapa* genes are illustrated with a color code at the bottom. **e** Gene connectivity characterization for singletons, retained pairs and retained triplicates in *B. rapa*. The color code reflects the nature of the considered genes (singletons, duplicates and triplicates) with regard to their physical location: LF (*red*), MF (*green*) and MF2 (*blue*). The *grey bar* (*right*) corresponds to the remaining specific genes from *B. rapa* not considered in the previous classes

The lineage-specific hexaploidization of *B. rapa*, leading to three subgenomes (LF, MF1 and MF2), has been the subject of intense studies and speculation (reviewed by Cheng et al. [16]). Our study revealed that the extant *B. rapa* genome derived from an n = 7 Calepineae ancestor (called “PCK” in the literature) with 21,035 ordered protogenes (from a total of 37,227 protogenes identified between *B. rapa* and *T. parvula*). The PCK evolved from ABK (eight protochromosomes covered by 20,037 ordered protogenes) through two reciprocal translocations (between ABK6 and ABK8) and one translocation (between ABK5 and ABK6/8) followed by one inversion on the newly formed ABK5/6/8, deleting the paleo-centromere of ABK8 (Fig. 3a, pink chromosome) and, finally, a reciprocal translocation between ABK2 and ABK5/6/8 (Fig. 3a, top).

The n = 7 PCK was then triplicated (hexaploidization), followed by fusion and fission events to reach the ten extant *B. rapa* chromosomes containing 39,498 genes. Fig. 3a (bottom left dot plot) shows the conservation between the ten *B. rapa* (x-axis) and seven PCK (y-axis) chromosomes with a color code illustrating the ABK1–8 protochromosomes. The detailed *B. rapa* paleohistory established that one paleo-centromere was lost between ABK and PCK on ABK8 (Fig. 3a, top, pink chromosome), through a translocation between ABK6 and ABK8, then fused to ABK5, leading to the monocentric ABK5-6-8 neochromosome. From the 21 post-hexaploidization PCK paleo-centromeres, we observed that the modern *B. rapa* centromeres derived from either a single paleo-centromere (for *B. rapa* chromosomes 1, 3, 4, 5, 6, 7, and 10 deriving from PCK 2, 2, 7, 1, 5, 3, and 6) or from the fusion of two retained ancestral centromeres (for *B. rapa* chromosomes 2, 8, and 9 deriving from PCK 5–6, 1–7, and 2–6) (Fig. 3a, bottom left dot plot, black circles). The remaining paleo-centromeres (one from PCK 1, 5, and 7; two from PCK 3; three from PCK 4) have been lost though pericentric inversions and translocations (Fig. 3a, bottom left dot plot, gray circles), as illustrated in the previous section for *A. thaliana* chromosome 1 (Fig. 2e).

Precise reconstruction of the PCK ancestor also offered the opportunity to investigate the number of PCK protogenes retained on the triplicated fragments in *B. rapa.* Brassiceae subgenomes (LF, MF1, MF2) have generally been investigated based on orthologous relationships, mostly with *A. thaliana* as a diploid representative of the *B. rapa* diploid ancestral karyotype [14, 15]. We re-assessed biases in ancestral gene retention since *B. rapa* derived from PCK and we found 506 protogenes retained as triplets and 7634, 3972 and 2395 retained as singletons in LF, MF1 and MF2 compartments, respectively (Fig. 3a, bottom; Table S4). Fig. 3a (bottom right dotplot) illustrates differences in ancestral gene retention (with a color code illustrating LF in red, MF1 in green and MF2 in blue) observed in the triplicated regions of the ten *B. rapa* chromosomes (x-axis). We reinvestigated the ‘two-step’ evolutionary theory [12] based on superimposed subgenome dominances by measuring retention of genes for 100-gene windows covering our reconstructed PCK chromosomes 1–7 (y-axis). MF1 and MF2 progenitors have been proposed in this theory to have become respectively dominant (D, reduced gene loss) and sensitive (S, enhanced gene loss) after a first hybridization event, and LF supradominant compared with MFI and MF2 as sensitive following a second hybridization event (Fig. 3a, middle). This gives a ranking of subgenome sensitivity forces in the extant *B. rapa* genome with MF2 (which underwent two rounds of sensitivity, making this compartment the most fractionated) > MF1 (which underwent opposite dominance/sensitivity in the frame of the hexaploidization event) > LF (considered as supradominant, making this compartment the least fractionated one) (Fig. 3a, middle). Overall, the inferred structure of the ancestral PCK genome provides new evidence for such a two-step evolutionary model between three progenitors (MF1, MF2 and LF) with a first hybridization between MF1 and MF2, followed by a second hybridization between MF1–MF2 and LF leading to the allohexaploid progenitor of the modern *B. rapa* genome.

We exploited the reconstructed PCK genome to investigate the chronology of such hexaploidization and diploidization events. According to the two-step theory, consisting of fusion of a diploid (LF) with a tetraploid (MF1–MF2) resulting in a hexaploid, two genomes (in our case MF1 and MF2) would have evolved concomitantly in the same nucleus for a longer period of time than the third (i.e., LF). If the observed difference in ancestral gene retention between LF, MF1 and MF2 reflects the two-step evolutionary model, MF1 and MF2 would have evolved separately from LF and may share reciprocal shuffling events absent from LF. To test this hypothesis we precisely investigated exchange of dominance/sensitivity (suggesting recombination events) between 100-genes windows covering the PCK chromosomes. Fig. 3b illustrates dominance/sensitivity exchanges observed on PCK6, showing ten large blocks of dominance/sensitivity exchange (black vertical arrows), possibly reflecting recombination events between MF1 (i.e., *B. rapa* chromosome 2) and MF2 (i.e., *B. rapa* chromosome 3), in contrast to a single recombination event observed between LF (i.e., *B. rapa* chromosome 10) and MF1 (red vertical arrow). Structural divergence at the gene level between LF, MF1 and MF2 is also observed in Fig. 3c by a comparison between distributions of silent substitution rates (Ks) for gene pairs between LF, MF1 and MF2 subgenomes where the following Ks ranking is expected from the youngest to the oldest: LF–MF1 > MF1–MF2 > LF–MF2. Mutiple Ks peaks are observed when comparing all gene duplicates (blue distribution curve and arrows) that can be associated with LF–MF1 (red distribution curve and arrow), LF–MF2 (green distribution curve and arrow) and MF1–MF2 (purple distribution curve and arrow) paralogs. Overall, the observed structural divergence, both at the gene level (i.e., low gene conservation with higher Ks values between MF1 and MF2 subgenomes) and, more interestingly, at the chromosome level (i.e., higher recombination events between MF1 and MF2 subgenomes) suggests an independent origin of LF and MF subgenomes and then single hybridization events between them (MF1–MF2 followed by MF1/MF–LF) in deriving *B. rapa*.

The inferred ancestral PCK genome allowed us to investigate the ontology of genes that have been preferentially retained in triplets in *B. rapa* after the hexaploidization event (Fig. 3d). When comparing the Gene Ontology (GO) categories associated with ancestral retained genes (Fig. 3d, left) with those associated with retained triplicated genes (Fig. 3d, right), structural molecule activity, transferase activity, transporter activity, binding and transcription regulators appear to be molecular functions resistant to the fractionation process (i.e., genes retained as pairs after WGD, *p* values <0.05). It has been proposed that gene dosage relations, maintained in stoichiometric equilibrium after a WGD due to functional redundancies between duplicated copies, are lost when one of the duplicates is deleted. This mechanism was first described in yeast and human [29] and the model refined through studies in *Arabidopsis*, maize and *Drosophila* [30]. Fates of duplicated genes in this scenario are considered through their roles in macromolecular complexes or networks [31]. Diploidization-resistant genes are considered to be those which are dosage-sensitive and whose products are involved in protein–protein interactions (referred to as ‘connected genes’ in networks such as transcription regulators) and their deletion or a modification of their product concentration will impact or unbalance the whole network. In contrast, diploidization-sensitive genes are considered to be dosage-insensitive, corresponding to functions involved in more stable processes which do not involve complex machinery, and which may return to a singleton state after WGD. We tested the impact of the gene product connectivity within networks as a driving force of ancestral gene retention as duplicates after WGD, using as a proxy the number of connections reported in the public *B. rapa* interactome (see “Materials and methods”). Fig. 3e shows that more than 50 % of ancestral PCK genes retained as pairs (between LF and MF1, LF and MF2 or MF1 and MF2) are connected with at least one other gene product in networks, with an increase of up to 70 % of connected genes in networks for protogenes retained as triplicates (between LF, MF1 and MF2). In contrast, ancestral genes which have returned to a singleton state (either in LF, MF1 or MF2) appear less connected within the investigated networks. Our data support the dosage balance hypothesis as a driving force of the post-polyploidization fractionation where genes for which their product is highly connected within networks may be preferentially conserved as duplicates/triplicates.

Overall, bias in gene functions and gene product connectivity within networks is observed between diploidization-resistant and -sensitive genes in favor of the gene dosage hypothesis as a driving mechanism of the observed subgenome dominance between LF, MF1 and MF2 subgenomes deriving from the allohexaploidization of three PCK progenitors through a two-step evolutionary model.

### Integration of the referenced ABC system into our evolutionary ancestral karyotype system

**Fig. 4:**
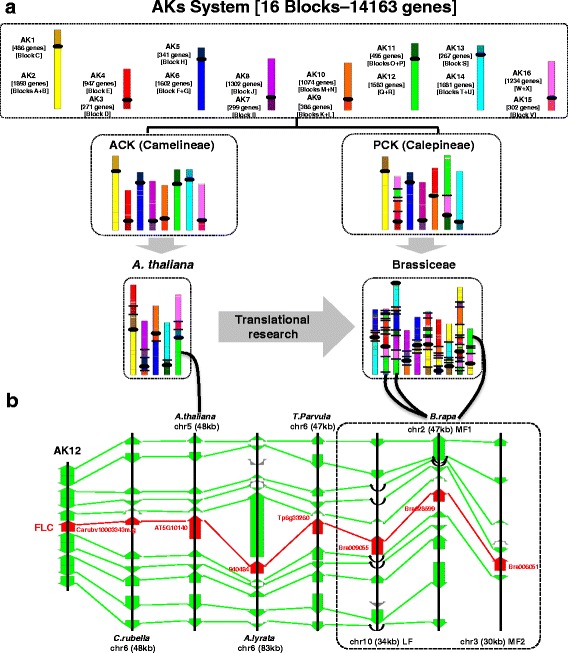
A new ancestral karyotype (*AK*) system for translational research in Brassicaceae. **a** Synteny between Camelineae (*A. thaliana* as extant representative) and Calepineae (*B. rapa* as extant representative) based on 24 ABC blocks (referred to as blocks A–X; adapted from Schranz et al. [6]) and 16 AK blocks (AK1–16 from the current analysis). The order, orientation and color-coding of the ABC and AK blocks are based on their position in ABK as illustrated in Fig. 1. **b** Microsynteny at the FLC locus between *A. thaliana*, *C. rubella*, *A. lyrata*, *T. parvula and B. rapa* based on the ancestral block AK12

The ABC nomenclature of syntenic blocks in Brassicaceae has been investigated since 2006 based on 24 conserved chromosomal blocks initially identified through the comparative genetic maps and comparative chromosome painting of *A. thaliana* and *B. rapa* [6] using BAC pools and has been proven to be extremely powerful for describing syntenic relationships between genomes. These blocks thus reflect the combined lineage-specific shuffling rearrangement that took place in both *A. thaliana* (from ABK to the five extant *A. thaliana* chromosomes) and in *B. rapa* (from ABK to PCK and to the ten extant chromosomes) (Fig. 4a, left). Consequently, this approach exaggerates the fragmentation of synteny relationships compared with the current paleogenomics study in which extant Brassicaceae genomes can now be simply presented as a mosaic of the eight ABK protochromosomes (instead of 24 GBs). It should be noted, however, that the ABC system is purely an operational set of conserved blocks from pairwise comparisons and therefore has no specific biological meaning, as no functional or evolutionary processes have been shown to be associated with these blocks. In addition, the ABC system has little meaning outside the Brassicaceae, because outgroups show direct syntenic relationships with the n = 8 ABK that either merge or split ABC blocks when using *A. thaliana* as a pivot (Fig. 1). Here, we simply integrated the ABC nomenclature into a coherent ancestral karyotype (AK) representation that is both consistent with the ABC nomenclature and meaningful from a broader evolutionary perspective that considers Brassicaceae as well as outgroups.

We thus integrated the ABC system (24 GBs) into our evolutionary AK system whose 16 blocks (corresponding to the long and short arms of ABK1–8) are covered by 14,163 genes conserved in all the investigated species. The correspondence between the AK (evolutionary set of ancestral blocks) and ABC (operational set of conserved blocks) systems is provided at the block level (Fig. 4a) as well as at the gene level (Table 1; Additional files 1, 2, 3 and 4). For any Brassicaceae of interest the AK system allows (*i*) robust identification of sets of orthologous gene pairs, (*ii*) generation of complete sets of chromosome to chromosome (or 16 blocks) relationships within and between genomes, and (*iii*) provision of a list of ordered protogenes within protochromosomes that can be used as a matrix for efficient marker development, genetic and physical map construction, annotation improvement and, ultimately, translational research from models to crops (Table 1). Indeed, efficient translation of structural genomic information from *A. thaliana* to *B. rapa* can be obtained from the current paleogenomics data and associated AK system, as illustrated by the example of the *FLC* locus (Fig. 4b). *FLC* is a MADS-box gene that drives the vernalization response in late-flowering ecotypes of *A. thaliana*. Our paleogenomics data provide the evolutionary scenario of the *FLC* locus since the ABK. It is clear from this that the *FLC* is an ancestral gene located on ABK6 that has been conserved in the extant species (*C. rubella* chromosome 6, *A. thaliana* chromosome 5, *A. lyrata* chromosome 6, *T. parvula* chromosome 6) and retained in three copies in *B. rapa* following the hexaploidization event [32]. This example illustrates the immediate access to robust orthologous relationships between species and the identification of putative functional orthologs in *B. rapa* from a candidate gene in *A. thaliana*.

Overall, the current study defines the n = 8 ABK in terms not only of large-scale chromosome architecture but also precise ancestral gene content (20,037 ordered protogenes based on 36,892 conserved genes) and provides a new AK standard system for the scientific community based on 16 conserved ancestral genomic blocks (covered by 14,163 genes conserved in all the investigated species). This will facilitate the translational research-based inference of candidate genes from models such as *A. thaliana* to Brassiceae crop species.

## Discussion and conclusions

### Reconstructed ancestral progenitors reveal the reticulate evolution of extant Brassicaceae

Previous phylogenetic and CCP investigations reported ancestral Brassicaceae karyotypes such as ABK (n = 8), PCK (n = 7) and translocated PCK (tPCK, n = 7), with ABK closely related to the extant *A. lyrata* and *C. rubella* genomes [6, 7, 33]. Up to now the ancestral Brassicaceae genome consisted of eight blocks of genes conserved between pairs of extant species, divided into 24 GBs [9]. In the current study these ancestral genomes have been entirely reconstructed for the Brassicaceae (ABK structured in eight chromosomes with 20,037 ordered protogenes based on a total set of 36,892 protogenes), Camelineae (ACK structured in eight chromosomes with 22,085 ordered protogenes based on a total set of 30,968 protogenes) and Calepineae (PCK structured in seven chromosomes with 21,035 ordered protogenes based on a total set of 37,227 protogenes), refining previous low resolution comparative genomics and genetics studies. The most recent study aimed at investigating the ancestral crucifer karyotype using a catalog of *A. thaliana* genes (alternatively blocks) putatively spanning the eight ancestral chromosomes [8]. The inferred ABK reconstruction we present here includes 30,968 protogenes based on conservation of gene adjacencies observed between five extant Brassicaceae genomes so that only 93 % (25,359 genes in ABK out of 27,206 annotated genes) of the modern *A. thaliana* gene repertoire can be considered as ancestral. The current data therefore represent a significant advance in understanding Brassicaceae evolution compared with published studies by considering plant genome evolution in general, and Brassicaceae evolution in particular, from the standpoint of ancestral genomes as a paradigm shift compared with current practice that has so far relied on pairwise comparisons of extant genomes or used a single pivotal or model genome such as *A. thaliana*.

These reconstructed progenitors allow us to refine the evolutionary events (chromosome fusion, fission, translocation as well as gains versus losses of genes, functions, repeats, etc.) and assign them to each specific lineage or species. In this context, genome rearrangements in the course of history appear to have been more active in particular compartments (i.e., centromeres, telomeres, duplication/inversion hotspots) so that evolutionary plasticity is partitioned in the extant Brassicaceae genomes. Following the polyploidization reported in *B. rapa* [12], the return to a diploid status (i.e., diploidization) took place by biased deletion of ancestral duplicated genes, leading to dominant (less fractionated, LF in *B. rapa*) and sensitive (more fractionated, MF1 and MF2 in *B. rapa*) subgenomes [8, 14–16]. Considering the fate of PCK triplicated genes, the bias in gene functions and gene product connectivity within networks observed between diploidization-resistant and -sensitive genes is in favor of the gene dosage hypothesis as a driving mechanism of subgenome dominance between LF, MF1 and MF2. More interestingly, the fate of triplicated PCK genes in post-polyploidization homoeologous compartments in *B. rapa* at the block (between LF, MF1 and MF2) and gene (Ks analysis of duplets/triplets) levels is consistent with the allohexaploid (i.e., referenced two-step theory [14, 15]) origin of *B. rapa* from three closely related PCK progenitors. In this scenario MF subgenomes deriving from the first tetraploidization evolved concomitantly for a longer period of time than LF subgenomes derived from the second hybridization event. Ancestral PCK genes within MF subgenomes are therefore more structurally related to each other than to homoeologous LF genes.

Overall, through ancestral genome reconstruction, we observed that the evolutionary plasticity is partitioned in the Brassiceae genomes with labile fractions corresponding to transposable element-rich (centromeric) and dynamic (duplication/inversion hotspots such as telomeres) loci in extant diploids and more fractionated blocks in extant paleopolyploids as a consequence of the diploidization phenomenon.

### Paleo-centromere loss as a major mechanism driving extant Brassicaceae evolution

Comparison of the ABK, ACK and PCK ancestors with the extant Brassicaceae species allowed the investigation of the fate of paleo-centromeres, either retained as extant functional centromeres or lost in the process of chromosome number reduction from ABK to present-day Brassicaceae. Chromosome number reduction from ABK (n = 8) to *A. thaliana* (n = 5) led to three centromere losses though pericentric inversion followed by reciprocal translocation [13]. Fine analysis of the extant *A. thaliana* chromosome 1, created by the fusion of ABK1 and ABK2, clearly established that only a single pericentromere, acting as the extant *A. thaliana* chromosome 1 centromere and corresponding to paleo-centromere ABK1, can be detected on the basis of the repeat density. The second paleo-centromere (from ABK2) has been efficiently erased, leaving only regions of a few kilobases on the extant chromosome. Altogether, our work illustrates how a heterogeneous distribution of repeats can be interpreted in the light of karyotype history.

This region of inversion/translocation, leading to the paleo-centromere ABK2 loss, appears to be a locus of intense plasticity with high deletion and recombination rates. Firstly, the high rate of deletions implies that DNA loss in this region generates structural variants among individuals and populations on which selection applies, apparently towards overall size reduction. Secondly, our analysis of crossing over frequencies in different *A. thaliana* chromosome 1 chunks suggests that this compartment is a privileged region for recombination. Overall, these observations may provide new clues about the potential benefits of chromosome rearrangements, by impacting the mixing of alleles within and across populations.

From the 21 post-hexaploidization PCK paleo-centromeres [16], we established that seven act as extant centromeres in *B. rapa* chromosomes 1, 3, 4, 5, 6, 7, and10, six have been fused into *B. rapa* chromosomes 2, 8, and 9, and the remaining eight lost through pericentric inversion following reciprocal translocations. Our current analysis shows that, when comparing the reconstructed ancestral genomes with their extant diploid (i.e., ABK versus *A. thaliana*) or paleopolyploid (PCK versus *B. rapa*) representatives, a general mechanism of chromosome reduction via fusions is observed, driven by centromere loss through inversion and translocation.

Overall, paleo-centromeres are either conserved, fused into a neo-centromere or lost by inversion and translocation, thus driving major structural rearrangements during chromosome number reduction to produce functional monocentric extant chromosomes.

### Ancestral genomes for high-resolution translational research from model to crop genomes

Efficient transfer of information, such as candidate genes for traits of interest from model species to crops, mainly relies on the robust identification of functional orthologous genes between species. The current analysis provides ancestral genomes for the Brassicaceae (36,892 conserved genes), Camelineae (30,968 conserved genes), and Calepineae (37,227 conserved genes). It constitutes the most accurate repertoire of orthologous and paralogous relationships within Brassicaceae. Such a catalog can now be considered as a template to select orthologs from *Arabidopsis* in Brassiceae crops (such as *B. rapa*, *Brassica oleraceae*, *Brassica nigra*) for trait improvement, i.e., translational research.

Brassicaceae have been reported to share 24 GBs of collinear genes based on CCP and synteny analysis using *A. thaliana* as a pivotal genome compared with *B. napus*, *A. lyrata* and *C. rubella* [5, 6, 34]. These syntenic blocks are classically used in comparative genomics studies between Brassicaceae as representative of the ancestral chromosomes that generated present-day species. From the current paleogenomics investigation of the Brassicaceae, we deliver a new AK system consisting of crucifer building blocks that can be rearranged to model any extant Brassicaceae genomes as well as other crucifer species when additional genetics maps, cytogenetics and genomics data become available. Such an AK standard system integrating the operational ABC system (i.e., 24 GBs), which we propose for the scientific community based on 16 conserved ancestral genomic blocks (containing 14,163 highly transferable and conserved genes in all the investigated species), will also facilitate the translational research-based inference of candidate genes from models such *A. thaliana* to Brassiceae crop species.

Overall, the provided Brassicaceae evolutionary scenario and associated reconstructed ancestral genomes (ABK, ACK and PCK) can now be considered by the scientific community (*i*) as a standard system for comparative genomics analyses with future genome sequences and (*ii*) as a backbone in mapping expressional and epigenetics data to investigate accurately the evolutionary fate of transcriptomes and epigenomes in driving Brassiceae evolutionary success through polyploidization.

## Materials and methods

### Genome sequences

*A. thaliana* (Arabidopsis Genome Initiative, 2000), *A. lyrata* [9], *C. rubella* [10], *T. parvula* [11], and *B. rapa* [12] draft genome sequences were downloaded from Phytozome [35] (http://www.phytozome.net/). For these extant species, we downloaded all the protein-coding sequences, together with their gene location and gene family name from the EnsemblGenome Compara database [36, 37].

### CAR identification

The EnsemblAPI was used to extract sub-gene trees containing at least one protein from the species described above. We then integrated the proteins from species not included in EnsemblAPI using a methodology previously developed for vertebrate species [38]. Briefly, (1) we performed BLASTP comparisons between predicted protein sequences from each of the new species against the 18 species from EnsemblGenome, and filtered the results with a cutoff value of 10^e-04^, (2) we calculated the average family bitscores between a given family of protein sequences and each protein of the additional species to insert (if a protein was associated with more than one family, we chose the family with the highest average bitscore), and (3) we performed a multiple alignment using M-Coffee [39] and computed a new reconciled gene tree with the species tree using the Treebest pipeline [37]. We thus obtained families containing orthologous and paralogous plant genes which were used as input for the AGORA (Algorithm for Gene Order Reconstruction in Ancestor) program to infer the gene content of each ancestor as presented here. The order of the ancestral genes was computed independently for each ancestor. In a first step, for a given ancestor, pairwise comparisons of gene order were performed between all pairs of extant genomes connected by branches that cross the ancestral genome of interest (i.e., two direct descendants or a descendant and an outgroup). For example, to compute the gene order in ABK, 129 pairwise comparisons of extant species were computed and analyzed to infer the ancestral gene organization. Secondly, the pairwise comparisons were used to identify conserved gene adjacencies, which occur when two genes, a1 and b1 in species 1, are adjacent, and their respective orthologs, a2 and b2 in species 2, are also adjacent. We constrained this conservation of adjacencies by the fact that the two adjacent genes also conserved their transcriptional orientation. To deal with the fact that genes can be specific to certain lineages, AGORA first filters the two genomes to retain only genes present in their last common ancestor. The conserved gene pairs are then considered as potentially ancestral. In a third step, the common adjacencies are recorded and combined in a weighted graph where nodes represent genes and links between nodes the number of times the adjacency is conserved. At this stage, inconsistencies may appear in the form of ancestral genes connected to more than two neighbors. To resolve this, the graph is then processed using a top-down greedy algorithm where the links of highest weight are selected first and are used to select the most likely gene-to-gene adjacency in case of multiple choices and while avoiding cycles. The algorithm ends with a fragmented genome composed of linear paths for each ancestor (that we call contigs) connecting ancestral genes based on the number of times their respective descendants are observed as extant neighbors. After extraction of these linear paths for each ancestor, a second round of AGORA is performed, by comparing contig order instead of gene order. The algorithm thus compares adjacencies of contigs in each pairwise extant genome that is informative for an ancestor, and builds a graph of contig adjacencies. The graph is then linearized to obtain the final blocks (or scaffolds) of ancestral oriented gene order.

### CAR scaffolding

For ABK, ACK and PCK, AGORA scaffolds with more than 50 genes were manually super-scaffolded based on chromosome synteny relationships between ancestral and extant genomes. For ABK, 20 scaffolds containing 20,037 genes (out of 461 scaffolds of minimum two genes) were super-scaffolded into eight chromosomes. For ACK, ten scaffolds containing 22,085 genes (out of 279 scaffolds of minimum two genes) were super-scaffolded into eight chromosomes and, finally, for PCK, 21 scaffolds containing 21,035 genes (out of 930 scaffolds of minimum two genes) were super-scaffolded into seven chromosomes for the reconstruction. All the karyotype reconstructions can be visualized and compared in a dedicated graphical browser [40] at http://genomicus.biologie.ens.fr/genomicus-plants-16.03/.

### Inference of gene gain and loss

In order to get the net gene gain of ACK and PCK, we computed the number of ACK and PCK genes that were not present in ABK. The net gene gain is the number of genes in *A. thaliana*, *A. lyrata*, *C. rubella* and *B. rapa* and *T. parvula* that were not present in ACK and PCK. For the net gene loss, we computed ABK genes that do not exist in our reconstructed ACK and PCK ancestors. Finally the net gene loss in extant species corresponds to ACK and PCK genes that are not present in each extant species. The lineage- or even species-specific genes in *C. rubella* were considered in 100-kb sliding windows based on *C. rubella* genes that are ABK protogenes which are not among the ordered (20,037 protogenes) or the total set of protogenes (36,892 protogenes).

### Regular and deep repeatome inference

The *C. rubella* deep repeat annotation was performed as described previously for *A. thaliana* [41]. Briefly, consensus sequences representative of repeated elements present in the *C. rubella* genome were constructed using TEdenovo from the REPET pipeline [41]. These sequences were used as probes to annotate the *C. rubella* genome with TEannot from the REPET pipeline [41]. The copies identified in the genome were then used in two ways: i) those bigger than 200 bp were used as probes to run TEannot a second time on the genome; ii) those of at least 100 bp and 75 % identity to the cognate consensus sequence were used as input to run P-clouds using 15-mers and the suite of parameters 2, 4, 3, 6, 12. The annotations obtained were combined to calculate repeat density.

### LF, MF1 and MF2 identification in the *B. rapa* genome

Using as reference the seven PCK ancestral chromosomes, considered as the pre-triplication ancestor, we identified LF, MF1 and MF2 regions of the *B. rapa* genome based on the number of ancestral genes retained in 100-gene windows. For each region of 100 consecutive PCK genes, three regions are identified in *B. rapa*. The three regions are then referenced as LF (region with more genes, i.e., reduced ancestral gene deletion), MF2 (with the fewest genes, i.e., highest ancestral gene deletion) and MF1 for the intermediate (remaining region).

### Network analysis

The public *B. rapa* interactome [42] was downloaded and we computed for each class of gene copies (LF, MF1, MF2, LF–MF1, LF–MF2, LF–MF1–MF2, *B. rapa*-specific genes) the proportion of genes that are connected with at least one partner within networks.

### Gene ontology and Ks analysis

Using AgriGO software (http://bioinfo.cau.edu.cn/agriGO/), we identified GOs that are significantly (false discovery rate adjusted *p* value ≤0.05) and differently represented in all ancestral retained genes, and in LF–MF1–MF2 genes compared with the genes in the entire genome. The representation was generated using revigo software (http://revigo.irb.hr/). The size of blocks corresponds to the significance in terms of the false discovery rate of the different GO. Ks calculation was performed using the reference PAML package [43, 44].

## Additional files

Additional file 1: Table S1. ABK (eight chromosomes and 36,892 genes) gene repertoire. (XLS 11537 kb) Additional file 2: Table S2. ACK (eight chromosomes and 30,968 genes) gene repertoire. (XLS 7922 kb) Additional file 3: Table S3. PCK (seven chromosomes and 37,227 genes) gene repertoire. (XLS 7818 kb) Additional file 4: Table S4.
*B. rapa* subgenome (LF, MF1 and MF2) repertoire. (XLS 3130 kb)

## Abbreviations

ABK: ancestral Brassicaceae karyotype
ACK: ancestral Camelineae karyotype
AK: ancestral karyotype
BAC: bacterial artificial chromosome
CAR: contiguous ancestral region
CCP: comparative chromosomal painting
D: dominant
GB: genomic block
GO: Gene Ontology
LF: least fractionated
MF: most fractionated
PCK: proto-Calepineae karyotype
S: sensitive
WGD: whole genome duplication
