# Erratum to: Understanding Brassicaceae evolution through ancestral genome reconstruction

**DOI:** 10.1186/s13059-016-0887-2

**Published:** 2016-04-04

**Authors:** Florent Murat, Alexandra Louis, Florian Maumus, Alex Armero, Richard Cook, Hadi Quesneville, Hugues Roest Crollius, Jerome Salse

**Affiliations:** INRA/UBP UMR 1095 GDEC ‘Génétique, Diversité et Ecophysiologie des Céréales’, 5 Chemin de Beaulieu, Clermont Ferrand, 63100 France; Ecole Normale Supérieure, Institut de Biologie de l’ENS, IBENS, Paris, F-75005 France; Inserm, U1024, Paris, F-75005 France; CNRS, UMR 8197, Paris, F-75005 France; INRA UMR 1164 URGI Route de Saint Cyr, Versailles, 78026 France; CNRS/UPVD UMR 5096 LGDP, 58 avenue P. Alduy, Perpignan, 66860 Cedex France

After the publication of this work [1] an error was noticed in Figure 1. There was an extra blue bar added in panel a in the bottom row far left. The corrected Figure 1 has now been updated in the original article. The publisher apologises for this error.
